# Comment on “Comparison of Diaphragmatic Stretch Technique and Manual Diaphragm Release Technique on Diaphragmatic Excursion in Chronic Obstructive Pulmonary Disease: A Randomized Crossover Trial”

**DOI:** 10.1155/2020/7437019

**Published:** 2020-05-18

**Authors:** Bruno Bordoni

**Affiliations:** Foundation Don Carlo Gnocchi IRCCS, Department of Cardiology, Institute of Hospitalization and Care with Scientific, Via Capecelatro 66, Milan 20100, Italy

I have read with interest the article by Nair and colleagues and I congratulate them for their publication [1]. The study employs patients with COPD (20 patients) and manual techniques to relax and lengthen the diaphragm muscle.

From the reading of the text emerge some critical issues that I would like to highlight.

The first is the technique that is used to lengthen the diaphragm muscle: the approach is based on maintaining an inspiratory attitude, by pulling the operator's hands down on the patient's ribs. This is a mistake. When the diaphragm performs an act of inhalation, the musculature shortens and does not lengthen; this happens on an animal model and on a human model [2–5]. The technique is based on a wrong principle. The lengthening of the diaphragm occurs during the expiratory act.

In patients with COPD, the diaphragm is in an attitude of inspiration and tries to maintain a further position of inspiration; very probably, it is not the most correct solution [6].

The technique is performed on seated and slightly inclined patients. The respiratory innervation of the diaphragm involves medullary nuclei (pre-Botzinger and parafacial retrotrapezoid) and the retroambiguus nucleus of the bulb [7].

During an act of inhalation, these centers of the breath activate the retrusion of the tongue, the lowering of the diaphragm, and the activation of the abdominal muscles and the pelvic floor [7].

The seated position does not facilitate a correct descent of the pelvic floor, altering an adequate inhalation, while the slight forward inclination of the trunk slows down the diaphragm's descent because the back musculature is put in tension, which is in contact with the diaphragm muscle [7, 8]. The article does not describe the distribution in the working groups: how many men and how many women in each group; which FEV1 of the patients in each group; and the age of patients for each group. The calculations that the authors carry out cannot be compared, because the description of each group is missing.

We know that ultrasound highlights the results that are operator-dependent [3]. The study does not describe who performed the ultrasound examination and when and if it was the same operator with all patients.

In the article references, there are two books in Spanish (25, 26). Not only are the books not recognized as medical texts but other researchers and scientists who want to deepen their research without the knowledge of Spanish cannot elaborate.

I believe that the article has several weak points which do not help the search, nor the clinical practice.
